# The -786T>C promoter polymorphism of the *NOS3 *gene is associated with prostate cancer progression

**DOI:** 10.1186/1471-2407-8-273

**Published:** 2008-09-29

**Authors:** Karina Marangoni, Thaíse G Araújo, Adriana F Neves, Luiz R Goulart

**Affiliations:** 1 Federal University of Uberlândia, Institute of Genetics and Biochemistry, Molecular Genetics Laboratory, Campus Umuarama, Block 2E, Room 24, 38400-902, Uberlândia, MG, Brazil

## Abstract

**Background:**

There is no biological or epidemiological data on the association between *NOS3 *promoter polymorphisms and prostate cancer. The polymorphisms in the promoter region of *NOS3 *gene may be responsible for variations in the plasma NO, which may promote cancer progression by providing a selective growth advantage to tumor cells by angiogenic stimulus and by direct DNA damage.

**Methods:**

This study aimed evaluating the *NOS3 *promoter polymorphisms by PCR-SSCP and sequencing, associating genotypes and haplotypes with *NOS3 *expression levels through semi-quantitative RT-PCR, and with *PCA*3 mRNA detection, a specific tumor biomarker, in the peripheral blood of pre-surgical samples from 177 patients; 83 PCa and 94 BPH.

**Results:**

Three novel SNPs were identified -764A>G, -714G>T and -649G>A in the *NOS3 *gene promoter region, which together with the -786T>C generated four haplotypes (N, T, C, A). *NOS3 *gene expression levels were affected by the -786T>C polymorphism, and there was a 2-fold increase in *NOS3 *levels favored by the incorporation of each C allele. *NOS3 *levels higher than 80% of the constitutive gene expression level (*B2M*) presented a 4-fold increase in PCa occurrence.

**Conclusion:**

The -786T>C polymorphism was the most important promoter alteration of the *NOS3 *gene that may affect the PCa progression, but not its occurrence, and the incorporation of the C allele is associated with increased levels of *NOS3 *transcripts. The *NOS3 *transcript levels presented a bimodal behavior in tumor development and may be used as a biomarker together with the *PCA3 *marker for molecular staging of the prostate cancer.

## Background

Enzymes responsible for nitric oxide (NO) synthesis constitute a family with at least three distinct isoforms, inducible, neuronal and endothelial [1]. The nitric oxide synthase 3 (*NOS3*) is located at the 7q35-q36 chromosome locus and the characterization of the 5'-flanking genomic region indicates that the *NOS3 *promoter is 'TATA-less'. This feature has been described for genes such as housekeeping genes and is usually associated with multiple transcription start sites. The NO synthase mRNA does not correspond to these criteria and the presence of specific potential transcription factor binding sites in the promoter could account for the cell specificity of its transcription [2].

The *NOS3 *seems to have an important role in vascular development, maintenance of the vascular tone and tumor growth in human prostate cancer (PCa) [3]. The net effects of NO depend on its available concentration, target cell, and interactions with reactive oxygen species (ROS), metal ions, and proteins [4,5].

The tumor-associated NO production may promote cancer progression by providing a selective growth advantage to tumor cells, by the angiogenic stimulus [6,7] and by the raise on mutations due to direct action of free radicals in DNA [8], and may also stimulate hyperplasia in normal tissue [8-10].

**Figure 1:**
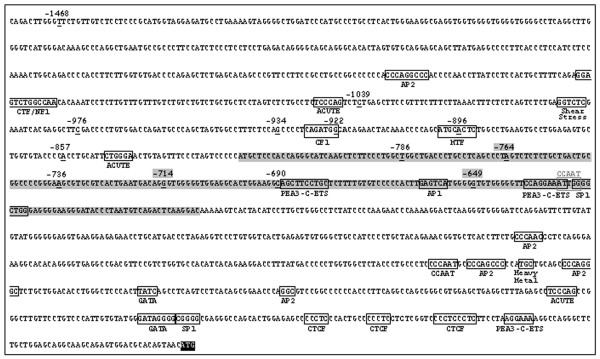
**Nucleotide sequence of *NOS3 *gene promoter**. Potencial cis-regulatory sequences are indicated in white boxes. The translation iniciation codon ATG is indicated in a black box. Underlined-bold nucleotides are indicating the 13 point mutations described for the promoter region. The three novel mutations and the 236-bp amplified fragment are highlighted in gray boxes.

Genetic polymorphisms in the promoter region of *NOS3 *gene (Figure 1) may be responsible for variations in the genetic control of plasma NO [11,12]. Although the promoter region is not part of the mRNA, it is directly related to the exons, and might be intervening at gene expression, decreasing or increasing it. Moreover, mutation points present in 5'- flanking region may confer a higher instability to the mRNA. To date ten variant *NOS3 *alleles have been identified in this region, these are, or may be, associated with decreased enzyme activity [13].

In this study, we have performed a specific analysis of a 236 base pair (bp) fragment of the *NOS3 *gene promoter region flanking the polymorphisms -786T>C and -690C>T, through DNA conformational assays (SSCP – single strand conformation polymorphism) and sequencing, determining genotypes and haplotypes, and estimating their possible association with PCa and benign prostatic hyperplasia (BPH) diseases. Three novel single nucleotide polymorphisms (SNPs) were characterized. Additionally, we have evaluated the effect of these five *NOS3 *SNPs on the mRNA expression levels of the peripheral blood of PCa and BPH patients, and their association with circulating tumor cells in the blood.

## Methods

### Patients, sample collection and preparation

This work was developed at the Laboratory of Nanobiotechnology of the Federal University of Uberlandia (UFU) together with the Urology Service of the University Clinics' Hospital. All the peripheral blood samples were obtained from patients that live in Uberlandia – MG (Brazil) and were enrolled during 2003 and 2004. The ethnic background was not recorded since the Brazilian population is highly heterogeneous and miscegenated, so it cannot be determined. The investigation was approved by the UFU Research Ethics Committee under the number 005/2001. Peripheral blood samples were collected before surgery in a vacutainer™ tube containing K_2 _EDTA 7.2 mg, and maintained at 4°C. To search for possible mutations in the promoter region of the *NOS3 *gene, we have performed the PCR-SSCP analysis in blood samples from 177 patients, which were grouped into two classes: 83 PCa (mean age, 69 years; range, 50 to 87 years) and 94 BPH (mean age, 68 years; range, 49 to 87 years), according to histological classification of tissues. BPH patients were submitted to TURP, except five patients that have undergone open prostatectomy. All PCa patients were submitted to radical prostatectomy, and were selected by using the following criteria: negative X-rays and bone scan analyses, and rectal examination compatible with organ confined cancer. DNA was extracted from leukocytes according to protocol previously published [14]. The mRNA was isolated by the Guanidine Isothiocyanate extraction method [15] with minor modifications. DNA and mRNA concentrations and quality were obtained by using the spectrophotometric absorbance readings at 260 and 280 nm.

### Amplification and genotyping of the -786T>C polymorphism

The presence of the -786T>C polymorphism in the 5'- flanking region of the *NOS3 *gene was determined by PCR amplification with the primers 5'-ATG CTG CCA CCA GGG CAT CA- 3' and 3'-GTC CTT GAC TCT GAC ATT AGG G- 5' [16]. A volume of 30 μL was used for each PCR reaction, which contained 5 ρmoles of each primer, 200 μM of each dNTP (desoxyribonucleotide triphosphate), 1.5 mmol/L MgCl_2_, 50 mmol/L KCl, 10 mmol/L Tris-HCl at pH 8.3 and 1 U Taq DNA polymerase (Phoneutria) with 4 μL of genomic DNA. The conditions of amplification were denaturing at 95°C-5 min, 35 cycles at 94°C-1 min, 62°C-1 min, 72°C-1 min, and a finally termination at 72°C-10 min. The amplified fragments were separated on 1.5% agarose gel electrophoresis and stained with ethidium bromide.

### PCR-SSCP analysis

A PCR-SSCP analysis, described elsewhere [17], was performed to detect mutations within the *NOS3 *promoter region. Two microliters of PCR products were mixed with 18 μL of a low ionic strength buffer (10% sucrose, 0.01% bromophenol blue, and 0.01% xylene cyanol) and heat-denatured for 10 min at 97°C. Products were separated on 15% polyacrylamide gel electrophoresis (49:1 – acrylamide:bisacrylamide) with 200 volts for 19 hours at room temperature, and detected by silver nitrate staining [18,19] with minor modifications.

### Purification of SSCP fragment bands and sequencing

**Figure 2:**
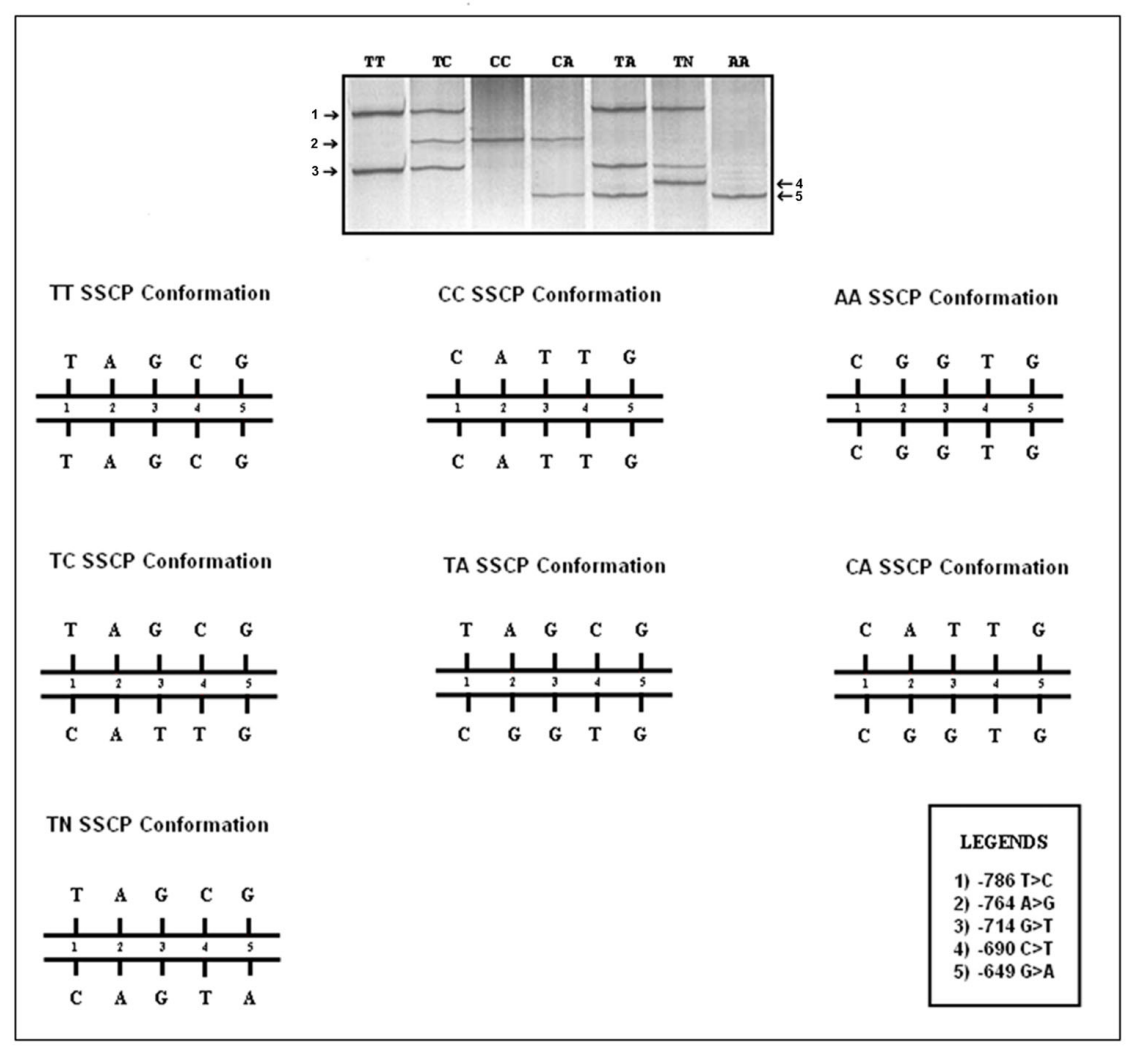
**PCR-SSCP analysis**. 15% polyacrylamide gel electrophoresis (49:1 acrylamide:bisacrylamide) with silver staining, evidencing the seven genotypic conformations and their chromosome models with four possible haplotypes, constructed based on the five *NOS3 *promoter polymorphisms investigated.

During genotyping of the -786T>C polymorphism of the *NOS3 *gene promoter region, three novel SNPs have been discovered [-764A>G, GenBank: RefSeq NM_EF042808/-714G>T, GenBank: RefSeq NM_EF042809/-649G>A, GenBank: RefSeq NM_EF042810]. Additionally, the two SNPs previously described [-786T>C, GenBank: RefSeq NM_2070744/-690C>T, GenBank: RefSeq NM_3918225] were also characterized. Seven SSCP conformations were observed in the polyacrylamide gel electrophoresis (Figure 2), although only three conformations were expected.

Each DNA fragment band originated from the PCR-SSCP technique was scrapped and individually reamplified using the same PCR reaction condition.

The purification of reamplified fragments was performed by DNA precipitation with ammonium acetate (7.5 M) and the pellet was ressuspended in 10 μL of water. DNA concentration and quality were obtained by using the spectrophotometric absorbance readings at 260 and 280 nm.

The purified products were submitted to a capillary sequencer (MegaBace 1000), using the DYEnamic ET Dye Terminator Cycle Sequencing kit (GE Healthcare). Three sequencing reactions had been carried for each fragment and injected twice to minimize sequencing artifacts. Sequences were processed and edited in the software DNASTAR Lasergene – SeqMan and EditSeq – (version 7.0, 2006). Sequences were aligned with the corresponding *NOS3 *sequence (GenBank: NM_348236) using the MegAlign procedure (DNASTAR Lasergene). The consensus sequences for each SSCP fragment band was established and comparisons were performed based on the original *NOS3 *gene sequence.

### RNA extraction and RT-PCR

Two microgramas of total RNA from blood, 10 U of RNase inhibitor (Invitrogen), 40 U of the *Murine Moloney Leukemia Virus *Reverse Transcriptase (MMLV-RT) (Amersham Biosciences), 1X MMLV-RT Buffer, 200 μM of each dNTP and 6 μM of hexamer random primers were incubated at 37°C for 1 hour and heated at 95°C-5 min. The 20 μL final volume of each reaction was completed with DEPC (diethylpyrocarbonate)-treated water. For normalization of amplification reactions, the internal positive control gene, the constitutive *B2M *gene (*B2M*: 5' -AGC AGA GAA TGG AAA GTC AAA- 3' and 5' -TGT TGA TGT TGG ATA AGA GAA- 3'), generating a 534-bp fragment, was used to validate reactions and to further characterize RNA quality of each sample.

### Nested RT-PCR for the *PCA3 *transcript detection

**Figure 3:**
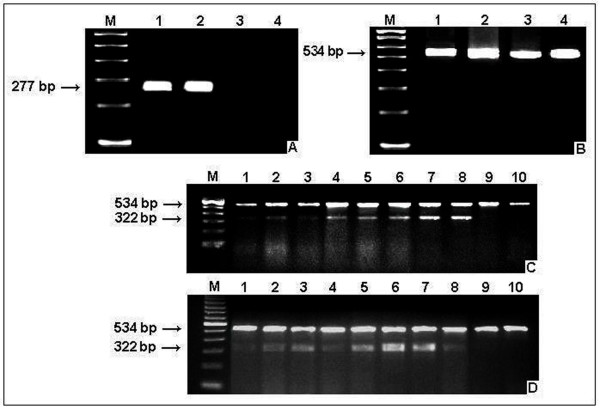
**Nested RT-PCR for the *PCA3 *and *B2M *gene expression analysis**. A) Lanes 1 to 4: amplifications for the *PCA3 *gene, where sample pairs 1 and 2, 3 and 4, correspond to two RNA extraction replicates for each patient. The detection of the expected 277-bp *PCA3 *fragment evidences the presence of circulating tumor cells. Lane M: 100-bp size marker. B) Lanes 1 to 4: amplifications for the *B*2*M *gene (534-bp). Semi-quantitative RT-PCR for the *NOS3 *and *B*2*M *gene expression analysis. The 322-bp and 534-bp fragments correspond to the *NOS3 *and *B*2*M *genes, respectively. Lane M: 100-bp size marker. Lanes 1 to 10 represent individual patients, where (C) represent BPH patients' profile, and (D) the PCa patients' profile.

A sensitive nested PCR assay for the detection of *PCA3 *mRNA was performed as previously reported [20] with minor modifications. A total of 137 patients were analyzed for the expression of circulating tumor cells in the peripheral blood, as evidenced by the *PCA3 *tumor biomarker positivity (Figure 3A and 3B).

### Semi-quantitative RT-PCR of the *NOS3 *gene

A representative sample of the genotyped population was selected, consisting of 38 patients (18 PCa and 20 BPH cases), and were analyzed in three replicates for the *NOS3 *mRNA relative expression levels. PCR reactions consisted of: 4 μL of cDNA, 1 U of Platinum Taq DNA Polymerase (Invitrogen), 50 mmol/L KCl, 10 mmol/L Tris-HCl at pH 8.3, 200 μM of each dNTP, 8 ρmoles of each oligonucleotide, 2 mmol/L MgCl_2_. The reaction was incubated for 48 cycles at 94°C-40 s, 55°C-40 s, 72°C-50 s, preceded by an initial denaturation at 95°C-2 min and a final extension cycle at 72°C-10 min. The designed primers sequence for the *NOS3 *were: sense 5' -CCT CAG GTT CTG TGT GTT CG- 3' and antisense 5'- GAT CAG ACCT GGC AGC AAC T- 3', generating a 322-bp fragment. The *B2M *constitutive gene was concomitantly amplified in the same reaction as described before.

### Densitometric readings to estimate relative gene expression levels

The amplicons obtained for both *NOS3 *and *B2M *genes were analyzed and quantified according to their agarose signal intensities by using the ImageMaster VDS Software program version 2.0 (Amersham Biosciences). The densitometric readings were normalized by using the target *NOS3 *mRNA:*B2M *mRNA ratio and relative levels were estimated for each sample (Figure 3C and 3D).

### Statistical analysis

Chi-square analyses were performed to compare genotypic and haplotypic frequencies for the average of clinical parameters, such as: age, PSA serum levels. Multiple regression analysis and Pearsons' correlations were used to verify the association among genotypes, *PCA3 *detection and clinical data. The Shapiro-Wilk test was used to verify normality of the relative levels of *NOS3 *gene expression, and mean comparisons of the mRNA relative levels were performed between PCa and BPH patients' groups through the Mann-Whitney test. Probability levels below 5% (p < 0.05) were considered significant. A cut-off value was determined for the *NOS3 *transcript relative levels and the OR were estimated to verify the chance of PCa occurrence in the presence of high concentrations of *NOS3 *transcripts. Pearsons' correlation analysis was performed among *NOS3 *relative expression levels of PCa and BPH patients in association with patients' age, serum PSA levels, the TNM adenocarcinoma histopathological staging, gleason score and polymorphisms.

## Results

### Molecular characterization of the SSCP conformations and the haplotypes models of the promoter region

**Figure 4:**
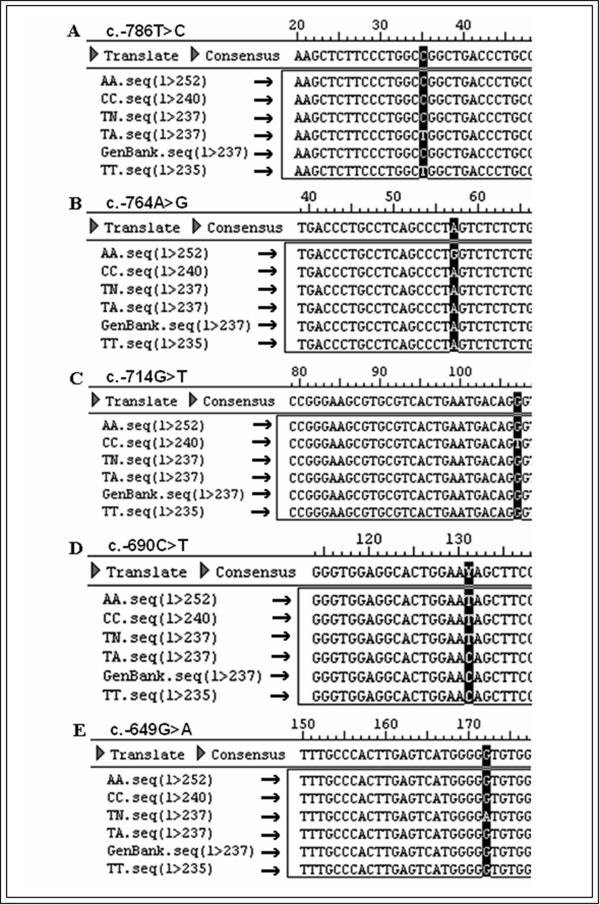
**Sequences alignment using DNASTAR Lasergene (SEQMAN and EDITSEQ), version 7.0 (2006)**. The point mutations are represented in a black box. A) -786T>C polymorphism. B) -764A>G polymorphism. C) -714G>T polymorphism. D) -690C>T polymorphism. E) -649G>A. Coding DNA reference sequence of promoter *NOS3 *gene [GenBank: NM_348236]. SSCP Conformations: AA, CC, TN, TA and TT.

Sequences of the SSCP electrophoretic bands were obtained and the consensus sequence was established (Figure 4). Sequence alignments were compared with the original sequence of the *NOS3 *gene [GenBank: NM348236], and all SNPs were positioned in the sequence, and their mutation classifications were assigned (Table 1).

**Table 1:** *NOS3 *gene promoter point mutations according to site and type.

**Position within promoter region**	**Position within 236-bp *NOS3 *fragment**	**Mutation classification**	**Nucleotide base codes (IUPAC)**	**dbSNP ID (GenBank)**
- 1468	--	Transition	W	not deposited
- 1038	--	Transversion	S	NM_2243311
- 976	--	Transversion	V	NM_2243311
- 934	--	Transversion	S	NM_2243311
- 922	--	Transition	R	NM_2243311
- 896	--	Transition	Y	NM_2243311
- 857	--	Transition	Y	NM_3918161
- 786	35	Transition	Y	NM_2070744
**(ŧ) **- 764	57	Transition	V	NM_EF042808
- 736	83	Insertion	N	NM_35886277
**(ŧ) **- 714	107	Transversion	K	NM_EF042809
- 690	131	Transition	Y	NM_3918225
**(ŧ) **- 649	172	Transition	V	NM_EF042810

**(ŧ) **novel single nucleotide polymorphism

**(--) **polymorphism located outside the 236-bp

**(W) **A or T; **(S)**G or C; **(V)**A or C ou G; **(R)**A or G; **(Y)**C or T; **(N)**any base; **(K)**G or T.

Based on consensus sequences, it was possible to construct four specific haplotypes N (-786T>C; -690A>G; -649G>A), C (-786T>C; -714G>T; -690C>T), A (-786 T>C; -764A>G; -690C>T) and T (no mutations). Chromosome models were also generated to explain the seven SSCP electrophoretic conformations: TT (no mutations), TC (-786T>C + [=], -714G>T + [=], -690C>T + [=]), CC (-786T>C + -786T>C, -714G>T + -714G>T, -690C>T + -690C>T), TA (-786T>C + [=], -764A>G + [=], -690C>T + [=]), AA (-786T>C + -786T>C, -764A>G + -764A>G, -690C>T + -690C>T), CA (-786T>C + -786T>C, -764A>G + [=], -714G>T + [=], -690C>T + -690C>T and TN (-786T>C + [=], -690C>T + [=], -649G>A + [=]) (Figure 2).

### SSCP conformations and haplotypic frequencies of the five mutations in promoter region of *NOS3 *gene

Table 2 shows SSCP conformations with their respective genotypes and haplotypic frequencies for all five characterized polymorphisms performed in 177 patients, from which 83 (46.9%) were histologically diagnosed as PCa and 94 (53.1%) as BPH. The -786T>C polymorphism presented the highest frequencies in both patients' groups, with higher frequencies for the TT and TC SSCP conformations. For the PCa group, all the other conformations (TA, CA, TN, and AA) did not present frequencies higher than 6%; however, the same four genotypic conformations presented higher frequencies in the BPH group, with 13.8% (13 out of 94) for the TA SSCP conformation, and 9.6% (9 out of 94) for the CA SSCP conformation.

**Table 2:** *SSCP *conformation and haplotypic frequencies, clinical parameters and laboratory data of the *NOS3 *gene in peripheral blood of patients with prostate cancer and benign prostatic hyperplasia.

		**PCa (N = 83)**							**BPH (N = 94)**				
**SSCP Conformation**	**Genotypes^(a)^**	**N (%)**	* Age	* PSA **(ng/ml)**	* TNM score	* Gleason score	**% positive ***PCA3*	*ŧ [*NOS3*]	**% (N)**	* Age	* PSA **(ng/ml)**	**% positive** *PCA3*	*ŧ [*NOS3*]

**TN**	c. [-786T>C] + [=] c. [-690C>T] + [=] c. [-649G>A] + [=]	1 (1.2)	77	101.0	--	9	0 (0 out of 1)	--	0	--	--	--	--
**CC**	c. [-786T>C] + [-786T>C] c. [-714G>T] + [-714G>T] c. [-690C>T] + [-690C>T]	9 (10.8)	67.5 (7.3)	41.4 (78.8)	1	8	42.9 (3 out of 7)	--	8 (8.5)	66.8 (7.8)	15.3 (11.7)	12.5 (1 out of 8)	0.5 (0.2)
**TT**	No mutations	30 (36.2)	68.5 (8.4)	24.0 (28.4)	2	7	28.0 (7 out of 25)	1.1 (1.8)	29 (30.9)	66.7 (9.2)	9.8 (10.3)	18.5 (5 out of 27)	0.5 (0.3)
**TC**	c. [-786T>C] + [=] c. [-714G>T] + [=] c. [-690C>T] + [=]	32 (38.6)	69.7 (7.8)	17.3 (18.7)	1	6	35.7 (10 out of 28)	1.1 (1.5)	35 (37.2)	68.9 (8.4)	11.9 (12.3)	15.4 (4 out of 26)	0.6 (0.4)
**TA**	c. [-786T>C] + [=] c. [-764A>G] + [=] c. [-690C>T] + [=]	4 (4.8)	64.5 (10.0)	11.6 (7.2)	2	6	66.7 (2 out of 3)	4.7 (4.3)	13 (13.8)	67.2 (10.1)	9.5 (7.3)	20.0 (2 out of 10)	0.4 (0.3)
**CA**	c. [-764A>G] + [=] c. [-714G>T] + [=] c. [-786T>C] + [-786T>C] c. [-690C>T] + [-690C>T]	5 (6.0)	63.8 (7.4)	9.4 (3.4)	1	6	40.0 (2 out of 5)	6.0 (0.3)	9 (9.6)	66.7 (8.6)	11.9 (7.9)	42.9 (3 out of 7)	0,6
**AA**	c. [-786T>C] + [-786T>C] c. [-764A>G] + [-764A>G] c. [-690C>T] + [-690C>T]	2 (2.4)	70.5 (7.8)	12.1 (12.1)	--	7	50.0 (1 out of 2)	--	0	--	--	--	--
Total		83					43.1 (25 out of 71)		94			19.0 (15 out of 78)	
**p^(1)^**	**p = 0.0082**								**p = 0.0011**				
**p^(2)^**	**p = 0.10**												

	**Haplotypes**	**%**	* Age	* PSA **(ng/ml)**	* TNM score	* Gleason score	**% positive** *PCA3*	*ŧ [*NOS3*]	**%**	* Age	* PSA **(ng/ml)**	**% positive** *PCA3*	*ŧ [*NOS3*]

**N**	c. [-786T>C; -690A>G; -649G>A]	1.0	77	101.0	--	9	0.0	--	0	--	--	0	
**T**	No mutations	58.0	69.9	38.5	1	6	56.0	1.1	56.0	67.6	10.4	53.2	0.4
**C**	c. [-786T>C; -714G>T; -690C>T]	33.0	66.7	22.7	1	7	32.0	1.1	32.0	67.5	11.0	30.1	0.3
**A**	c. [-786T>C; -764A>G; -690C>T]	8.0	66.3	11.0	2	6	12.0	2.6	12.0	66.9	10.7	16.7	0.2
Total		100.0					100.0 (25)		100.0			100.0 (15)	

(ŧ) representative sampling of the population; (*) mean (± SD); (--) inapplicable data

(1) Comparison (genotypes) in the groups with probability levels obtained by chi-square tests

(2) Comparison (genotypes) between groups with probability levels obtained by Kruskal-Wallis test

(a) Genotypes correspond to the promoter NOS3 polymorphisms

No significant differences for SSCP conformations frequencies between patients' groups were observed (p = 0.10). However, the comparison of the SSCP conformations frequencies within the PCa patients (p = 0.0082) and within BPH patients (p = 0.0011) were significant different.

There was no haplotypic frequencies difference between groups (p > 0.05). However, inspection of haplotypes revealed that the C (-786T>C; -714G>T; -690C>T) and T (no mutation) haplotype was the most common one in both patients' groups. One rare haplotype N (-786T>C; -690A>G; -649G>A) has been observed in PCa patients (1.2%). Although not significant, the haplotype A (-786 T>C; -764A>G; -690C>T) was more frequent in BPH patients (12%) than in PCa (8%). Both haplotypes and SSCP genotypic conformation frequencies distribution were in Hardy-Weinberg equilibrium.

The estimated chance for cancer occurrence, considering the seven SSCP genotypic conformations among groups (PCa and BPH), were: TT + TC + CC + CA + AA *versus *(*vrs*) TA (odds ratio (OR) = 3.13, CI_95%_, 0.98 – 10.01), TT + CA *vrs *TA (OR= 2.99, CI_95%_, 0.89 – 10.05), CC *vrs *CA + TA (OR= 3.38, CI_95%_, 1.00 – 11.37), TT *vrs *CA + TA (OR= 3.10, CI_95%_, 1.25 – 7.72). All combinations of SSCP conformations were tested for the OR, but only the most significant ones were demonstrated. It was observed a 3.38-fold higher chance of having cancer when the CC SSCP conformation is considered in relation to CA + TA. A 3.10-fold higher chance of having cancer was obtained for the TT SSCP conformation in relation to CA + TA. The TT + TC + CC + CA + AA *vrs *TA and TT + CA *vrs *TA associations were close to significance (p < 0.10), and presented a relative risk of 3.13 and 2.99-fold higher chance of having cancer, respectively.

Table 3 shows the estimated chance for cancer occurrence, considering haplotypes and their number of copies between groups (PCa and BPH), and no significant ORs were obtained. The sample size was quite small for some haplotypes, but we presented the true haplotypic frequencies of copy numbers in the population investigated for the polymorphisms within the 236-bp region, and although the haplotypes were not significantly associated with prostate cancer, this is the first description of such variations in the *NOS3 *promoter.

**Table 3:** *NOS3 *gene promoter haplotypes, number of copies, and prostate cancer risk.

**Haplotypes^a^** **(SSCP Conformation)**	**Units**	**Zero Copies**	**One Copy**	***p-Value***	**Two copies**	***p-Value***
No mutations **(T)**	Controls/cases	17/16	48/36	0.73	29/30	1.0
	OR (95% CI)	1.00 (ref)	0.80 (0.36 – 1.79)		1.10 (0.47 – 2.58)	
[-786T>C; -714G>T; -690C>T] **(C)**	Controls/cases	42/36	44/37	0.92	8/9	0.81
	OR (95% CI)	1.00 (ref)	0.98 (0.52–1.83)		1.31 (0.46 – 3.76)	
[-786T>C; -764A>G; -690C>T] **(A)**	Controls/cases	72/71	22/11	0.13	0/2	--
	OR (95% CI)	1.00 (ref)	0.51 (0.23 – 1.12)		--	
[-786T>C; -690A>G; -649G>A]^b^ **(N)**	Controls/cases	94/82	0/1	--	0/0	--
	OR (95% CI)	1.00 (ref)	--		--	

(a) Alleles listed for dbSNP ID (GenBank) in 5' to 3' order: NM_2070744, NM_EF042808, NM_EF042809, NM_3918225, NM_EF042810.

(b) Haplotype that appear only once between groups (PCa and BPH).

CI: confidence interval/OR: odds ratio

(--) inapplicable data

In agreement with the Kruskal-Wallis test, significant differences between patients' groups for mean prostate specific antigen (PSA) were observed (p = 0.0048). Pearsons' correlation coefficients for genotypes *vrs *PSA (p = 0.02), genotypes *vrs *gleason score (p = 0.0195), age *vrs *PSA (p = 0.0412), age *vrs *gleason score (p = 0.0421), gleason score *vrs *Tumor-Node-Metastasis (TNM) score (p = 0.0021) and PSA *vrs *TNM score (p = 0.0189) within the PCa patients were significantly different. No significant differences among BPH patients were observed (Table 2).

### Analysis of the *PCA3 *transcript detection and association with polymorphisms

The prostate cancer antigen 3 (*PCA3*) transcript detection (Figure 3A) was performed in 149 patients (149 out of 177), from which 109 (73.2%) were negative and 40 (26.8%) were positive. Among *PCA3 *positive patients, 62.5% (25 out of 40) were histologically diagnosed as PCa and 37.5% (15 out of 40) as BPH. Among *PCA3 *negative patients, 42.2% (46 out of 109) were PCa and 57.8% (63 out of 109) were BPH (Table 4). Although patients' histological classification (PCa and BPH) were carefully analyzed, it is possible that transurethral resection of prostate (TURP) biopsies of BPH patients have been misdiagnosed, once biopsy procedures may not have reached tumor specific sites.

**Table 4:** *PCA3 *mRNA detection in the peripheral blood of PCa and BPH patients across genotypic SSCP conformations and their haplotypes.

		**Positive *PCA3 *mRNA** **(N = 40)**			**Negative *PCA3 *mRNA** **(N = 109)**		
**SSCP**	**Genotypes**	**PCa** N (%)	**BPH** N (%)	**Total**	**PCa** N (%)	**BPH** N (%)	**Total**
**TN**	[-786T>C] + [=] [-690C>T] + [=] [-649G>A] + [=]	0	0	**0/0**	1 (0.9)	0	**1/109** **(0.9)**
**CC**	[-786T>C] + [-786T>C] [-714G>T] + [-714G>T] [-690C>T] + [-690C>T]	3 (7.5)	1 (2.5)	**4/40** **(10)**	4 (3.7)	7 (6.4)	**11/109** **(10.1)**
**TT**	No mutations	7 (17.5)	5 (12.5)	**12/40** **(30)**	18 (16.5)	22 (20.2)	**40/109** **(36.7)**
**TC**	[-786T>C] + [=] [-714G>T] + [=] [-690C>T] + [=]	10 (25)	4 (10)	**14/40** **(35)**	18 (16.5)	22 (20.2)	**40/109** **(36.7)**
**TA**	[-786T>C] + [=] [-764A>G] + [=] [-690C>T] + [=]	2 (5)	2 (5)	**4/40** **(10)**	1 (0.9)	8 (7.3)	**9/109** **(8.3)**
**CA**	[-764A>G] + [=] [-714G>T] + [=] [-786T>C] + [-786T>C] [-690C>T] + [-690C>T]	2 (5)	3 (7.5)	**5/40** **(12.5)**	3 (2.7)	4 (3.7)	**7/109** **(6.4)**
**AA**	[-786T>C] + [-786T>C] [-764A>G] + [-764A>G] [-690C>T] + [-690C>T]	1 (2.5)	0	**1/40** **(2.5)**	1 (0.9)	0	**1/109** **(0.9)**
Total		**25/40** **(62.5)**	**15/40** **(37.5)**	**40/149** **(26.8)**	**46/109** **(42.2)**	**63/109** **(57.8)**	**109/149**

**SSCP**	Haplotypes	**PCa** **%**	**BPH** **%**		**PCa** **%**	**BPH** **%**	

N	[-786T>C; -690A>G; -649G>A]	0	0		1	0	
T	No mutations	56	53		61	59	
C	[-786T>C; -714G>T; -690C>T]	32	30		31	32	
A	[-786T>C; -764A>G; -690C>T]	12	17		7	9	

* *PCA3 *gene expression (PCa = 71/BPH = 78)

*PCA3 *positivity in all SSCP conformations and haplotypes were also shown in Table 4. It was expected a higher frequency of *PCA3 *positivity in the PCa group and a lower frequency of positivity in the BPH group, as observed, although not significant. It is interesting to mention that the *PCA3 *positivity in the PCa group for the TC + CC conformations was 2.6 times higher than the frequency observed in the BPH group (32.5% *vrs *12.5%, P = 0.02). On the other hand, CC + TC conformations frequencies were not significantly different between patients' groups (PCa = 20.2% *vrs *BPH = 26.6%) within the negative *PCA3 *detection class (Table 4). The TA + CA conformations presented significantly higher frequencies in the BPH (11%) than in the PCa group (3.6%) within the negative *PCA3 *class (P = 0.03).

In the overall, the *PCA3 *clinical parameters for PCa cell detection in the peripheral blood were: 35.0% of sensitivity (25 out of 71), and 81.0% of specificity (63 out of 78); however, it is important to emphasize that a positive result for PCa patients is not an indication of metastasis, and the *PCA3 *detection in BPH patients may indicate that they may have been misdiagnosed.

For the negative *PCA3 *detection (Table 4), all genotypic frequencies were higher among BPH patients, except for the TN SSCP conformation that presented only a negative result for one PCa patient.

Considering the haplotypic frequencies for the positive and negative *PCA3 *detection classes, there were no differences between patients' groups. However, the T haplotype (no mutations) was the most frequent one followed by the C (-786T>C; -714G>T; -690C>T) and A (-786 T>C; -764A>G; -690C>T) haplotypes. The A haplotype was more frequent in BPH than in PCa patients, for both positive and negative *PCA3 *classes, although it was not significant.

### Analysis of the *NOS3 *gene expression levels in association with polymorphisms

The semi-quantitative analyses of the *NOS3 *transcript levels and their association with polymorphisms and haplotypes were performed by selecting a representative sample of the population based on the genotypic frequencies and clinical parameters. The 38 patients used in this study presented the same genotypic frequencies and average values for the clinical parameters observed in the patients' population (177 patients).

The *NOS3 *relative levels related to the beta-2-microglobulin (*B2M*) in the peripheral blood did not follow a normal distribution (n = 38, p = 0.33). Although not significant, the mean *NOS3 *relative levels was four times higher in PCa in relation to BPH patients (ratio *NOS3 *mRNA/*B2M *mRNA: 2.23 and 0.50, respectively) (Figure 3B).

A cut-off value for the relative levels of *NOS3 *transcripts were established based on the maximum average levels observed for the BPH group. The cut-off value for negative results was below 0.8 for the *NOS3 *mRNA/*B2M *mRNA ratio, which means 80% of the observed value for the *B2M *transcript levels. Considering the cut-off value, it was observed a 4.0-fold higher chance (CI_95%_, 0.95 – 16.77; p = 0.12) of having cancer when the *NOS3 *expression levels were equal or higher than 0.8.

**Figure 5:**
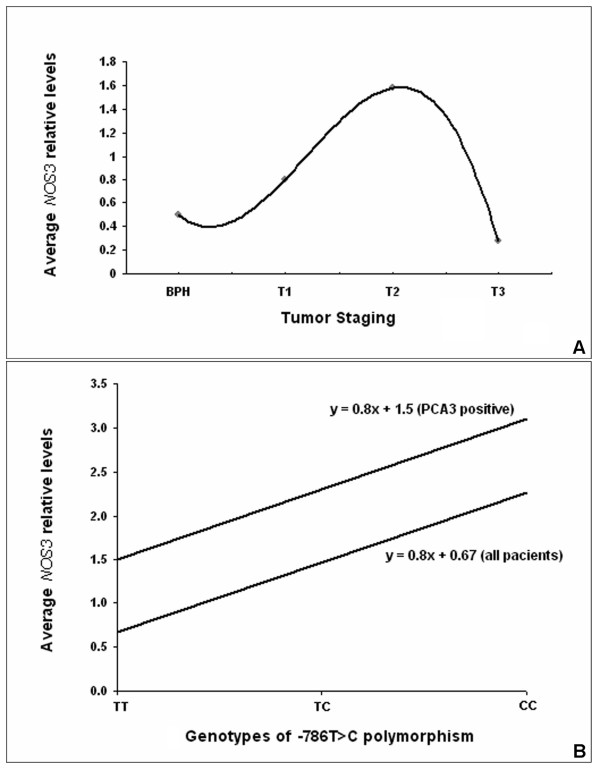
**Graphics representations**. A) The average *NOS3 *relative levels of PCa patients classified according to tumor stages and a predicted tendency line. B) Regression analysis for the average *NOS3 *relative levels in association with the -786T>C polymorphism genotypes for all patients and for the group of patients with positive *PCA3 *detection.

Among PCa patients, only the Pearsons' correlation coefficients obtained among the *NOS3 *gene expression and *PCA3 *data (p = 0.038) were significant. For BPH patients, the *NOS3 *levels, serum PSA, patients' age on diagnosis and *PCA3 *detection were not correlated among each other. However, the average *NOS3 *levels presented a bimodal behavior in PCa patients classified according to their tumor stages, with higher levels in the pT2 stage (Figure 5A).

No significant differences among *NOS3 *gene expression levels and haplotypes were observed between groups (Table 2). However, there was a significant association of *NOS3 *genotypes and gene expression levels for PCa patients (p = 0.011), but not within the BPH group. It was observed increased levels of *NOS3 *transcripts within the PCa group, especially for patients with TA and CA SSCP conformations, with *NOS3 *mRNA/*B2M *mRNA ratios of 4.7 and 6.0, respectively.

The average *NOS3 *levels were 3.52-fold higher in *PCA3 *positive patients (*NOS3 *mRNA/*B2M *mRNA = 3.17) in comparison to the average observed in *PCA3 *negative patients (*NOS3 *mRNA/*B2M *mRNA = 0.90), although not significant, due to the low number of *PCA3 *positive samples analyzed.

Analysis of the *NOS3 *expression levels across the -786T>C polymorphism on all patients showed a linear behavior with an additive component for each C allele incorporated into the genotype, with higher levels (2.27) observed for the (-786T>C + -786T>C) mutant homozygous patients, followed by medium levels (1.47) for the (-786T>C + [=]) heterozygous and lower levels (0.67) for normal homozygous patients (Figure 5B).

Similarly, the *NOS3 *levels within the positive *PCA3 *class (grouping BPH and PCa patients) presented a linear behavior considering the -786T>C polymorphism, with higher levels (3.1) for the (-786T>C + -786T>C) mutant homozygous patients, followed by medium levels (2.3) for the (-786T>C + [=]) heterozygous and lower levels (1.5) for normal homozygous patients (Figure 5B).

## Discussion

During the genotyping of the -786T>C polymorphism of the *NOS3 *promoter [16], we have discovered and characterized three novel mutations [-764A>G, GenBank: NM_EF042808/-714G>T, GenBank: NM_EF042809/-649G>A, GenBank: NM_EF042810] in the PCa and BPH patients. The association between these three mutations together with two SNPs previously described [-786T>C, GenBank: NM_2070744/-690C>T, GenBank: NM_3918225] and prostate cancer was further examined.

The five polymorphisms have generated seven genotypic profiles in the polymerase chain reaction-single-strand conformation polymorphism (PCR-SSCP) gel electrophoresis and four haplotypes, suggesting that some mutations are closely linked; therefore, they have evolved together, originating a restricted number of haplotypes. It is interesting to observe that when the -786T>C mutation was present at least two other mutations were linked. But, when the normal -786T allele was present, no additional polymorphisms were detected, explaining the four possible haplotypes.

This is the first publication that associates the promoter polymorphisms of the *NOS3 *gene with prostate cancer risk; however, other polymorphisms, such as the 894G>T (Glu298Asp) and the intron 4 have also been evaluated [21-24].

The possibility of linkage between the two previously described *NOS3 *gene polymorphisms and the promoter polymorphisms has not been investigated, and some controversies may occur in the literature probably due to the incorrect population stratification based on genotyping, which could be generating linkage disequilibrium among polymorphisms in the population that may present a functional association with the *NOS3 *expression and activity. However, there is an evidence that the 894G>T polymorphism had no influence on *NOS3 *transcriptional levels, but it was associated with *PCA3 *detection [24].

The strategy of determining genotypic conformations of the *NOS3 *promoter region through the SSCP technique demonstrated to be very effective in classifying all patients. The significant relative risks of cancer occurrence related to CC and TT SSCP conformation (3.38 and 3.10, respectively) in comparison to TA + CA suggest that the -786T>C polymorphism is the most important promoter alteration that may affect the PCa progression. It is also important to observe that patients with the CC SSCP conformation, in both PCa and BPH groups, presented the highest serum PSA averages.

In the present work, there is a good evidence that the -786T>C polymorphism affects the *NOS3 *expression, with the (-786T>C + -786T>C) mutant homozygous condition presenting the highest levels of *NOS3 *transcripts, and acting in an additive manner, as the C allele is incorporated into the genotype. This was also observed within the positive *PCA3 *detection class, although positive *PCA3 *patients present higher levels of *NOS3 *transcripts. However, some authors have associated the (-786T>C + -786T>C) genotype with low levels of *NOS3 *mRNA, which would be contributing to a higher risk of cardiovascular diseases [25-27].

The association of the -786T>C promoter polymorphism with *NOS3 *transcriptional levels and the highly significant odds ratio (4-fold) for elevated *NOS3 *levels (*NOS3*:*B2M *> 0.8) and the risk of PCa occurrence are important evidences of the *NOS3 *gene promoter role in the PCa progression. This is further supported by the 2-fold increase in *NOS3 *mRNA levels in the positive *PCA3 *detection class, as demonstrated in Figure 5B. The *NOS3 *transcriptional activity was also highly correlated with disease staging (Figure 5A), once its highest level is reached at the pT2 stage, when the angiogenic stimulus is required for tumor cell dissemination and metastasis.

Recent studies have described the NO involvement in many biological processes affecting carcinogenesis [28-31]. Our results are also supported by previous works [24], which have also indicated that *NOS3 *levels may present a bimodal behavior during cancer development.

The fine mapping of the *NOS3 *promoter region, flanking the region -820 to -583, has allowed us to construct four haplotypes and seven genotypic combinations. We have found that none of the haplotypes did affect significantly the *NOS3 *levels; in fact, a unique polymorphism (-786T>C) may be responsible for the *NOS3 *transcription regulation, and this polymorphism is observed only in haplotypes N, C and A.

The increasing *NOS3 *expression levels associated with the -786T>C polymorphism may contribute to cancer progression by providing a selective growth advantage of tumor cells, by the angiogenic stimulus [6,7] and by causing DNA damage due to the direct action of O_2_^- ^free radicals, as an effect of the excess NO production [8].

The suggestion that decreasing *NOS3 *expression levels, which consequently reduce the NO production, would have an anti-apoptotic role, and may promote tumor growth [32] may be explained in part by the bimodal behavior of the *NOS3 *levels across the stages as shown in this investigation and in a previous work [24], once lower levels is mainly seen in advanced tumor stages (pT3 and pT4).

The first evidence of the association of *NOS3 *polymorphisms with circulating tumor cells was demonstrated between the intron 4 polymorphism and the folate hydrolase – prostate-specific membrane antigen (*FOLH1*) expression in the peripheral blood [23]. According to these authors, patients with the 'a' allele have low plasmatic NO levels, and therefore are more inclined to have viable circulating tumor cells.

In this investigation, we did not find association between the *NOS3 *gene promoter polymorphisms with circulating tumor cells *PCA3 *detection in the peripheral blood, as shown elsewhere with the 894G>T polymorphism and the *PCA3 *detection [24].

The *PCA3 *gene is a highly specific prostate tumor biomarker that is not found in other kinds of cells and tissues, whereas its detection on peripheral blood may indicate a possible metastasis [20]. We believe that the *PCA3 *is more specific for tumor detection than the *FOLH1 *marker, which presents a high variation on gene expression among PCa and BPH patients and its utilization as a biomarker is highly controversial (unpublished data).

In the overall, the *PCA3 *clinical parameters for PCa cell detection in the peripheral blood were: 35.0% of sensitivity (25 out of 71), and 81.0% of specificity (63 out of 78); however, it is important to observe that a positive result is not an indication of metastasis and that some BPH cases may have been misdiagnosed, reducing its true clinical value.

In fact, the *PCA3 *detection may become a potential biomarker for blood diagnosis, once it is exclusively detected in prostate cancer cells; therefore, patients histologically diagnosed as BPH may probably be tumor confined disease that was missed during biopsy sampling or by pathological examination. On the other hand, a negative *PCA3 *detection in cancer patients may also suggest that the tumor is organ confined. However, it is important to emphasize that positive *PCA3 *detection is not an indication of faster tumor development or invasiveness.

Prostate cancer is a complex disease due to multifactorial and multifocal events caused by many biological mechanisms. These mechanisms are represented by differential gene expression profiles, generating disease developmental stages that vary from latent to aggressive forms. Therefore, detection of key genetic alterations at the molecular level may be a useful tool as a prognostic indicator. Despite the complexity of events that the NO participates in many biological processes, its association with carcinogenesis is critical once NO levels modulate tumor development. Part of this differential NO expression is regulated by *NOS3 *promoter polymorphisms, specifically the -786T>C, which may have an influence on the progression of the disease and not on its occurrence.

There are at least five regulatory elements within the amplified promoter region (-820 to -583) and their interaction with all polymorphisms have not been investigated. Therefore, it is possible that other external and internal factors may independently influence *NOS3 *gene expression masking the true effect of polymorphisms, which may explain the high variability of *NOS3 *transcript levels observed within PCa and BPH groups.

In the present work, higher *NOS3 *transcript levels in peripheral blood of positive *PCA3 *patients than in negative ones suggest that tumor may be in a medium-late (pT2) stage of the disease and corroborates with the molecular approach for disease staging. Therefore, we propose that patients with differential expression/detection, such as high *NOS3 *and negative *PCA3 *may indicate pT1 stage, while low *NOS3 *and positive *PCA3 *may indicate pT3 or pT4 stages.

## Conclusion

In conclusion, this is the first publication that demonstrates an association of the *NOS3 *promoter region polymorphisms with prostate cancer progression. The strategy of determining genotypic conformations of the *NOS3 *promoter region through the SSCP technique demonstrated to be very effective in genotyping all patients, which were classified into four haplotypes and seven genotypic conformations. The C allele of the -786T>C polymorphism was always associated with at least two other mutations (haplotypes C, A and N), while the T haplotype (containing the T allele) had no mutations. Significant relative risks (> 3 fold) of cancer occurrence were related to the -786T>C polymorphism, and it is the most important promoter alteration that may affect the PCa progression, but not its occurrence. *NOS3 *transcript relative levels (*NOS3*:*B2M *> 0.8) were 4-fold higher in PCa than in BPH, and due to its bimodal behavior, the *NOS3 *levels may be used as a biomarker together with the *PCA3 *marker for molecular staging of the disease.

## Abbreviations

NO: nitric oxide; *NOS3*: nitric oxide synthase 3; PCa: prostate cancer; ROS: reactive oxygen species; bp: base pair; SSCP: single strand conformation polymorphism; BPH: benign prostatic hyperplasia; SNP: single nucleotide polymorphism; OR: odds ratio; *vrs*: *versus*; PSA: prostate specific antigen; TNM: tumor-node-metastasis; *PCA3*: prostate cancer antigen 3; TURP: transurethral resection of prostate; *B2M*: beta-2-microglobulin, PCR: polymerase chain reaction; *FOLH1*: folate hydrolase – prostate-specific membrane antigen; UFU: Federal University of Uberlandia; dNTP: desoxyribonucleotide triphosphate; MMLV-RT: *murine moloney leukemia virus *reverse transcriptase; DEPC: diethylpyrocarbonate; CNPq: National Counsel of Technological and Scientific Development; CAPES: Coordination of Perfectioning of Staff of Superior Level; FAPEMIG: Foundation of Support to the Research of the State of Minas Gerais.

## Competing interests

The authors declare that they have no competing interests.

## Authors' contributions

KM, TGA and AFN have equally contributed for the present study (research design, sample collection, processing, and analysis). LRG is senior author and wrote the paper with KM. The authors read and approved the final manuscript.

## Pre-publication history

The pre-publication history for this paper can be accessed here:


